# Supplementary figures and images for: Prevalence and incidence of diabetic retinopathy in patients with diabetes of Latin America and the Caribbean: A systematic review and meta-analysis
Source: PLoS One. 2024 Apr 4;19(4):e0296998. doi: 10.1371/journal.pone.0296998 (PMC10994322; doi:10.1371/journal.pone.0296998)

Supplementary material 8. Funnel plot of the overall prevalence of diabetic retinopathy in T1DM.


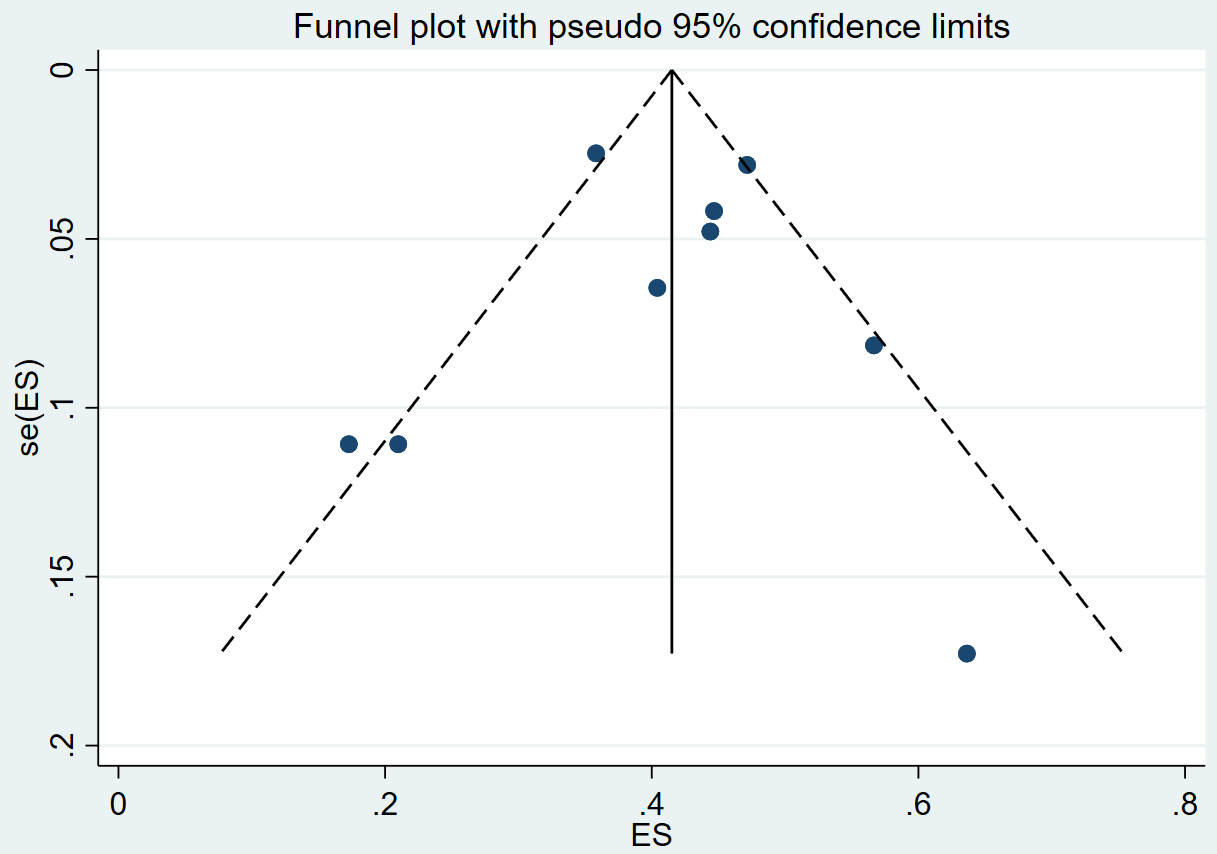

Supplement: S3 Fig — (DOCX) [file pone.0296998.s003.docx]
