# Supplementary material for: Prevalence and incidence of diabetic retinopathy in patients with diabetes of Latin America and the Caribbean: A systematic review and meta-analysis
Source: PLoS One. 2024 Apr 4;19(4):e0296998. doi: 10.1371/journal.pone.0296998 (PMC10994322; doi:10.1371/journal.pone.0296998)
Supplement: S4 Fig — (DOCX) [file pone.0296998.s004.docx]

Supplementary material 9. Funnel plot of the overall prevalence of diabetic retinopathy in T2DM.
