# Supplementary material for: Prevalence and incidence of diabetic retinopathy in patients with diabetes of Latin America and the Caribbean: A systematic review and meta-analysis
Source: PLoS One. 2024 Apr 4;19(4):e0296998. doi: 10.1371/journal.pone.0296998 (PMC10994322; doi:10.1371/journal.pone.0296998)
Supplement: S2 Table — (DOCX) [file pone.0296998.s006.docx]

## Supplementary material 2. Search Strategy in systematic review

Filter: None

Date of search: 16/01/2023

| Search strategy | Results |
| --- | --- |
| **Pubmed** |  |
| #1  Latin America[Mh] OR "Latin America"[tiab] OR Caribbean Region[Mh] OR "Caribbean Region"[tiab] OR South America [Mh] OR "South America”[tiab] OR Indians, South American [Mh] OR Hispanoamerica*[tiab] OR Iberoamerica*[tiab] OR Panamerican*[tiab] OR Argentina[Mh] OR Argentina[tiab] OR Argentin*[ad] OR Bolivia[Mh] OR Bolivia[tiab] OR Bolivia[ad] OR Brazil[Mh] OR Brazil[tiab] OR Brazil*[ad] OR Brasil*[ad] OR Chile[Mh] OR Chile[tiab] OR Colombia[Mh] OR Colombia[tiab] OR Colombia[ad] OR Costa rica[Mh] OR Costa rica[tiab] OR Costa Ric*[ad] OR Cuba[Mh] OR Cuba[tiab] OR Ecuador[Mh] OR Ecuador[tiab] OR Ecuador*[ad] OR El salvador[Mh] OR "El salvador"[tiab] OR "El salvador"[ad] OR Guatemala[Mh] OR Guatemala[tiab] OR Guatemala[ad] OR Haiti[Mh] OR Haiti[tiab] OR Honduras[Mh] OR Honduras[tiab] OR Mexico[Mh] OR Mexico[tiab] or Mexico[ad] or Mejico[ad] OR Nicaragua[Mh] OR Nicaragua[tiab] OR Panama[Mh] OR Panama[tiab] OR Paraguay[Mh] OR Paraguay[tiab] OR Paraguay[ad] OR Peru[Mh] OR Peru[tiab] OR Peru*[ad] OR Puerto Rico[Mh] OR "Puerto Rico"[tiab] OR "Puerto Rico"[ad] OR Dominican Republic[Mh] OR "Dominican Republic"[tiab] OR "Dominican Republic"[ad] OR Uruguay[Mh] OR Uruguay[tiab] OR Uruguay[ad] OR Venezuela [Mh] OR Venezuela [tiab] OR Venezuela [ad] OR Suriname[Mh] OR Suriname[tiab] OR Surinam*[ad] OR Guiana*[tiab] OR Guiana*[ad] OR Guyan*[tiab] OR Guyan*[ad] | 1188 |
| #2  “Diabetic Retinopathy” [MH] OR “Diabetic Retinopath*” [TIAB] OR “Diabetic Retinopath*” [OT] OR “diabetes mellitus retinopathy”[tiab] OR “diabetes retinopathy”[tiab] OR “diabetic retinitis”[tiab] OR “retinopathia diabetica” [tiab] OR “retinopathy, diabetes”[OT] OR “diabetes mellitus retinopathy”[OT] OR “diabetes retinopathy”[OT] OR “diabetic retinitis”[OT] OR “retinopathia diabetica” [OT]  OR (“Diabetes mellitus”[MH] AND “Retinal Diseases” [MH]) OR  (“Diabet*”[TIAB] AND (“Retinal Disease” [TIAB] OR  “Retinopath*”[TIAB] OR “Retinosis”[TIAB] OR “Retinitis”[TIAB])) OR (“Diabet*”[OT] AND (“Retinal Disease” [OT] OR “Retinopath*”[OT] OR “Retinosis”[OT] OR “Retinitis”[OT])) |  |
| #1 AND #2 |  |
| **Scopus** |  |
| #1  AFFILCOUNTRY ( argentina OR bolivia OR brazil OR brasil OR colombia OR chile OR ecuador OR guyana OR "french Guiana" OR paraguay OR peru OR suriname OR uruguay OR venezuela OR belize OR "costa rica" OR "el Salvador" OR guatemala OR honduras OR nicaragua OR panama OR mexico OR mejico OR cuba OR "dominican republic" OR haiti OR jamaica OR "Puerto rico" OR "trinidad and tobago" OR barbados OR guadeloupe OR grenada OR martinique OR bermuda OR bahamas ) | 1652 |
| #2  ( TITLE-ABS-KEY ( diabet*  W/3  retinopath* )  OR  TITLE-ABS-KEY ( diabet*  W/3  "Retinal Disease*" )  OR  TITLE-ABS-KEY ( diabet*  W/3  retinosis )  OR  TITLE-ABS-KEY ( diabet*  W/3  retinitis ) ) |  |
| #1 AND #2 |  |
| **Embase** |  |
| #1  'argentina':ca OR 'bolivia':ca OR 'brazil':ca OR brasil:ca OR 'colombia':ca OR 'chile':ca OR 'ecuador':ca OR 'guyana':ca OR 'french guiana':ca OR 'paraguay':ca OR 'peru':ca OR 'suriname':ca OR 'uruguay':ca OR 'venezuela':ca OR 'belize':ca OR 'costa rica':ca OR 'el salvador':ca OR 'guatemala':ca OR 'honduras':ca OR 'nicaragua':ca OR 'panama':ca OR 'mexico':ca OR mejico:ca OR 'cuba':ca OR 'dominican republic':ca OR 'haiti':ca OR 'jamaica':ca OR 'puerto rico':ca OR 'trinidad and tobago':ca OR 'barbados':ca OR 'guadeloupe':ca OR 'grenada':ca OR 'martinique':ca OR 'bermuda':ca OR 'bahamas':ca | 1143 |
| #2  (diabet* NEAR/3 retinitis):ti,ab,kw OR (diabet* NEAR/3 retinosis):ti,ab,kw OR (diabet* NEAR/3 'retinal disease*'):ti,ab,kw OR (diabet* NEAR/3 retinopath*):ti,ab,kw OR 'diabetic retinopathy-induced':de,ab,ti OR 'diabetic retinopathy':de OR 'diabetes mellitus retinopathy':tn,ti,ab OR 'diabetes retinopathy':tn,ti,ab OR 'diabetic retinitis':tn,ti,ab OR 'diabetic retinopathy':tn,ti,ab OR 'retinopathia diabetica':tn,ti,ab OR 'retinopathy, diabetes':tn,ti,ab |  |
| #1 AND #2 |  |
| **WoS** |  |
| #1  CU= (argentina OR bolivia OR brazil OR BRASIL OR colombia OR chile OR ecuador OR guyana OR "french Guiana" OR paraguay OR peru OR suriname OR uruguay OR venezuela OR belize OR "costa rica" OR "el Salvador" OR guatemala OR honduras OR nicaragua OR panama OR mexico OR mejico OR cuba OR "dominican republic" OR haiti OR jamaica OR "Puerto rico" OR "trinidad and tobago" OR barbados OR guadeloupe OR grenada OR martinique OR bermuda OR bahamas) | 1170 |
| #2  TS=(diabet* NEAR/3 retinopath*) OR TS=(diabet* NEAR/3 “Retinal Disease*”) OR TS=(diabet* NEAR/3 retinosis) OR TS=(diabet* NEAR/3 retinitis) |  |
| #1 AND #2 |  |
| **Scielo** |  |
| #1  CU= (argentina OR bolivia OR brazil OR BRASIL OR colombia OR chile OR ecuador OR guyana OR "french Guiana" OR paraguay OR peru OR suriname OR uruguay OR venezuela OR belize OR "costa rica" OR "el Salvador" OR guatemala OR honduras OR nicaragua OR panama OR mexico OR mejico OR cuba OR "dominican republic" OR haiti OR jamaica OR "Puerto rico" OR "trinidad and tobago" OR barbados OR guadeloupe OR grenada OR martinique OR bermuda OR bahamas) OR AD= (argentina OR bolivia OR brazil OR BRASIL OR colombia OR chile OR ecuador OR guyana OR "french Guiana" OR paraguay OR peru OR suriname OR uruguay OR venezuela OR belize OR "costa rica" OR "el Salvador" OR guatemala OR honduras OR nicaragua OR panama OR mexico OR mejico OR cuba OR "dominican republic" OR haiti OR jamaica OR "Puerto rico" OR "trinidad and tobago" OR barbados OR guadeloupe OR grenada OR martinique OR bermuda OR bahamas) | 284 |
| #2  TS=(diabet* NEAR/3 retinopath*) OR TS=(diabet* NEAR/3 “Retinal Disease*”) OR TS=(diabet* NEAR/3 retinosis) OR TS=(diabet* NEAR/3 retinitis) |  |
| #1 AND #2 |  |
| **Medline** |  |
| #1  TS=(diabet* NEAR/3 retinopath*) OR TS=(diabet* NEAR/3 “Retinal Disease*”) OR TS=(diabet* NEAR/3 retinosis) OR TS=(diabet* NEAR/3 retinitis) OR MH=(“Diabetic Retinopathy” OR (“Diabetes Mellitus” AND “Retinal Diseases”)) | 975 |
| #2  ad= (argentina OR bolivia OR brazil OR BRASIL OR colombia OR chile OR ecuador OR guyana OR "french Guiana" OR paraguay OR peru OR suriname OR uruguay OR venezuela OR belize OR "costa rica" OR "el Salvador" OR guatemala OR honduras OR nicaragua OR panama OR mexico OR mejico OR cuba OR "dominican republic" OR haiti OR jamaica OR "Puerto rico" OR "trinidad and tobago" OR barbados OR guadeloupe OR grenada OR martinique OR bermuda OR bahamas) |  |
| #1 AND #2 |  |
