# Supplementary material for: Prevalence and incidence of diabetic retinopathy in patients with diabetes of Latin America and the Caribbean: A systematic review and meta-analysis
Source: PLoS One. 2024 Apr 4;19(4):e0296998. doi: 10.1371/journal.pone.0296998 (PMC10994322; doi:10.1371/journal.pone.0296998)
Supplement: S3 Table — (DOCX) [file pone.0296998.s007.docx]

## Supplementary material 3. Excluded studies reviewed in full text

| Author - year | Title | Reason for exclusion |
| --- | --- | --- |
| Greca - 2012 | Clinical features of patients with type 2 diabetes mellitus and hepatitis C infection. | Classification not reported |
| Martinez - 2011 | Diabetic retinopathy screening using single-field digital fundus photography at a district level in Costa Rica: a pilot study. | Classification not reported |
| Menezes - 2020 | Fundoscopy findings of diabetic and/or hipertensive patients | Wrong population |
| Cádenas - 2019 | Influence of diabetes mellitus in the ocular surface | Wrong study design |
| Adrianzén - 2019 | Frequency and severity of diabetic retinopathy in patients with type 2 diabetes mellitus at the Regional Institute of Ophthalmology | Duplicate record |
| Flores - 2019 | Management of diabetic retinopathy using telemedicine and network integration | Duplicate record |
| Grassi - 2019 | Características clínicas, esquemas de tratamiento, grado de control y variables asociadas a este en una cohorte de adultos con diabetes mellitus tipo 1 en un centro terciario | Classification not reported |
| Hirakawa - 2019 | Knowledge of diabetic patients users of the Health Unic System about diabetic retinopathy | Wrong outcome |
| Milanés - 2019 | Incidence of ophthalmological affections in the Island of Fogo, Green Cape. 2015-2017 | Wrong population |
| Covarrubias - 2017 | Chile needs better statistics and greater coverage in screening for diabetic retinopathy | Wrong publication type |
| Rodríguez - 2017 | Prevalence of visual disability caused by diabetic retinopathy, a challenge to all | Wrong outcome |
| Faria - 2017 | Risk factors related to the intervention with intravitreal anti-VEGF injection in patients with diabetic macular edema | Wrong outcome |
| Esteves - 2008 | Fatores de risco para retinopatia diabetica. | Wrong publication type |
| Rodríguez - 2017 | Study on the prevention of visual disability in diabetic patients from Playa municipality | Wrong outcome |
| Covarrubias - 2017 | Coverage of a screening program and prevalence of diabetic retinopathy in primary cares | Duplicate record |
| Mendanha - 2016 | Risk factors and incidence of diabetic retinopathy | Wrong outcome |
| Hernández-Narváez - 2015 | Prevalence of visual disorders and their relationship with functionality of the older adults | Wrong outcome |
| Jiménez-Báez - 2015 | Early diagnosis of diabetic retinopathy in primary care | Wrong outcome |
| Barriga - 2020 | Using a Handheld Retinal Camera and Artificial Intelligence for Diabetic Retinopathy Screening in Bolivia | Wrong publication type |
| Zegada - 2013 | Diabetic Retinopathy and other ocular findings in diabetic patients, assisted by the Foundations Cristo Vive and Vida Plena, Bolivia | Wrong population |
| Dubán - 2012 | Diabetic retinopathy | Wrong publication type |
| Galiano - 2012 | Health condition and compliance of the chilean clinical guideline for type 2 diabetic patients | Classification not reported |
| Hernández - 2011 | Risk factors in the development of diabetic retinopathy | Wrong study design |
| Fernández - 2010 | Clinical-epidemiological behavior of ametropia | Wrong outcome |
| Dias - 2010 | Epidemiologic profile and level of knowledge among diabetic patients about diabetes and diabetic retinopathy | Wrong outcome |
| Llerena - 2010 | Main ophthalmologic diseases in Guanes municipality, Pinar del Río province | Wrong outcome |
| Grassi - 2019 | [Clinical features and management of 205 adults with type 1 diabetes mellitus]. FT Caracteristicas clinicas, esquemas de tratamiento, grado de control y variables asociadas a este en una cohorte de adultos con diabetes mellitus tipo 1 en un centro terciario. | Duplicate record |
| Guedes - 2009 | Prevalence of the diabetic retinopathy in a Family's Health Program unity | Classification not reported |
| Laclé-Murray - 2009 | Prevalence and Risk Factors of Diabetic Nephropathy in a Peripheral Urban Area of the Central Plateau of Costa Rica | Classification not reported |
| Scheffel - 2008 | The prevalence of chronic diabetic complications and metabolic syndrome is not associated with maternal type 2 diabetes | Classification not reported |
| Lisboa - 2008 | Relation between diabetic retinopathy and diabetic dermopathy in type 2 diabetes mellitus patients | Duplicate record |
| Licea - 2007 | Risk factors associated with the appearance of diabetic retinopathy | Wrong outcome |
| Cabrera - 2007 | Prevalence of blindness and severe visual shortage in over 50 years-old adults in the City of Havana province | Wrong outcome |
| Ramos - 2007 | Intravitreal triamcinolone to treat diabetic macular edema | Wrong study design |
| Bruron - 2019 | Low attendance to diabetic retinopathy screening in young people in England | Wrong publication type |
| Maia - 2007 | Delay in ophthalmologic examination of diabetic retinopathy patients | Wrong population |
| Hernández - 2006 | Resultados del RACSS en Ciudad de La Habana, Cuba, 2005 | Wrong population |
| Chacon - 2005 | Fundoscopic alterations and diabetic foot in patients of Hospital Universitário Onofre Lopes/UFRN | Classification not reported |
| Souza - 2004 | Diabetic retinopathy among patients assisted by a multidisciplinary program at the University Hospital of Ribeião Preto, Sao Paulo - USP | Duplicate record |
| Pereira - 2004 | The prevalence of diabetic retinopathy at the Pediatric Endocrinology Outpatient Clinic of the Santa Casa de São Paulo | Duplicate record |
| Garcia - 2003 | Diabetic retinopathy incidence and risk factors in patients of the Onofre Lopes University Hospital, Natal-RN | Classification not reported |
| Díaz - 2003 | Clinical Characteristics of diabetes mellitus type 2 in the municipality of Guines in the year 2002. | Classification not reported |
| Armengol - 2019 | Incidence of ophthalmological affections in the Island of Fogo, Green Cape. 2015-2017 | Wrong population |
| Barriga - 2018 | One Year Results of Clinical Use of an Automatic Diabetic Retinopathy Screening System at Diabetes Care Clinics | Wrong publication type |
| Almeida - 2017 | Micro and macro vascular complications in people with type 2 diabetes mellitus in outpatient care | Classification not reported |
| Barriga - 2017 | Automatic Diabetic Retinopathy Screening System at a Network of Comprehensive Diabetes Care Clinics in Monterrey, Mexico | Wrong publication type |
| Henao - 2017 | Frequency of visual impairment in the Diabetic Foot consult in high complexity hospital | Wrong outcome |
| Zamora - 2016 | Teleretinal Screening in Mexico: Automating Diabetic Retinopathy Screening at a Comprehensive Diabetes Care Clinic in Monterrey, Mexico | Wrong publication type |
| Zamora - 2014 | Teleretinal Screening in Mexico: Second Year of a Pilot Project at a Comprehensive Diabetes Care Clinic in Monterrey, Mexico and Preliminary Results on Clinical Outcome | Wrong publication type |
| Ramirez - 2010 | Effectiveness of Screening for Diabetic Retinopathy With Non-Mydriatic Camera in Latino Population | Wrong publication type |
| Luckie - 2004 | Identification and follow-up of diabetic retinopathy in rural health in Australian: an automated screening model | Wrong outcome |
| Hennis - 2002 | Incident diabetic retinopathy in the Barbados eye study | Wrong publication type |
| Veloso - 2020 | Predictors of sudomotor dysfunction in patients with type 1 diabetes without clinical evidence of peripheral neuropathy | Wrong outcome |
| Ferraz - 2020 | Portable devices in ophthalmology Reply: portable devices in ophthalmology Diabetic retinopathy screening and the COVID-19 pandemic in Brazil Diabetic retinopathy screening in urban primary care setting with a handheld smartphone-based retinal câmera The feasibility of smartphone based retinal photography for diabetic retinopathy screening among Brazilian Xavante IndiansThe English National Screening Programme for diabetic retinopathy 2003-2016 | Wrong publication type |
| Korn - 2020 | The feasibility of smartphone based retinal photography for diabetic retinopathy screening among Brazilian Xavante Indians | Classification not reported |
| Hainsworth - 2019 | Risk Factors for Retinopathy in Type 1 Diabetes: The DCCT/EDIC Study. | Wrong population |
| Salamanca - 2018 | Implementation of a diabetic retinopathy referral network, Peru. | Classification not reported |
| Cardoso - 2020 | Prognostic importance of visit-to-visit blood pressure variability for micro- And macrovascular outcomes in patients with type 2 diabetes- And Rio de Janeiro Type 2 Diabetes Cohort Study | Classification not reported |
| Zago - 2020 | Diabetic retinopathy detection using red lesion localization and convolutional neural networks | Wrong outcome |
| Joshi - 2020 | A retrospective study of causes of visual impairment and use of low vision devices in the low vision clinic in Trinidad and Tobago | Wrong population |
| Braithwaite - 2020 | National Eye Survey of Trinidad and Tobago (NESTT): Prevalence, causes and risk factors for presenting vision impairment in adults over 40 years | Wrong population |
| Menezes - 2020 | Fundoscopy findings of diabetic and/or hipertensive patients | Wrong population |
| Bergonsi - 2020 | Glycated Hemoglobin and Blood Pressure Levels in Adults With Type 2 Diabetes: How Many Patients Are on Target? | Classification not reported |
| Gomes - 2019 | Relationship between health care insurance status, social determinants and prevalence of diabetes-related microvascular complications in patients with type 1 diabetes: a nationwide survey in Brazil. | Duplicated population |
| Malerbi - 2020 | Retinal exams requested at Primary Care Unit: indications, results and alternative strategies of evaluation. | Classification not reported |
| Das - 2015 | Diabetic retinopathy: a global epidemic. | Wrong publication type |
| Chung - 2019 | Associations between serum apolipoproteins, urinary albumin excretion rate, estimated glomerular filtration rate, and diabetic retinopathy in individuals with type 2 diabetes | Classification not reported |
| Flores - 2019 | Management of diabetic retinopathy using telemedicine and network integration | Wrong outcome |
| Ovalle-Luna - 2019 | Prevalence of complications of diabetes and associated comorbidities in family medicine of the Mexican Institute of Social Security | Duplicate record |
| Grassi - 2019 | Clinical features and management of 205 adults with type 1 diabetes mellitus | Duplicate record |
| Melo - 2018 | Prevalence and risk factors for referable diabetic retinopathy in patients with type 1 diabetes: a nationwide study in Brazil | Wrong outcome |
| Farah - 2006 | Prevalence of retinopathy and microalbuminuria in pediatric type 2 diabetes mellitus. | Wrong population |
| Ferguson - 2010 | The epidemiology of diabetes mellitus in Jamaica and the Caribbean: a historical review. | Wrong publication type |
| Dodson - 1992 | Diabetes mellitus and retinal vein occlusion in patients of Asian, west Indian and white European origin. | Wrong population |
| Haffner - 1988 | Diabetic retinopathy in Mexican Americans and non-Hispanic whites. | Wrong country |
| Robinson - 2018 | High rates of ocular complications in a cohort of Haitian children and adolescents with diabetes | Classification not reported |
| Drummond - 2018 | Regional differences in the prevalence of diabetic retinopathy: A multi center study in Brazil | Duplicated population |
| Seferovic - 2018 | Retinopathy, neuropathy, and subsequent cardiovascular events in patients with type 2 diabetes and acute coronary syndrome in the ELIXA: The importance of disease duration | Wrong publication type |
| Cardoso - 2017 | Predictors of Development and Progression of Retinopathy in Patients with Type 2 Diabetes: Importance of Blood Pressure Parameters | Wrong outcome |
| Tres - 2007 | Prevalence and characteristics of diabetic polyneuropathy in Passo Fundo, South of Brazil. | Classification not reported |
| Vargas-Sánchez - 2011 | Coverage and results of a screening program for diabetic retinopathy using mydriatic retinography in primary health care. | Wrong outcome |
| Covarrubias - 2017 | Chile needs better statistics and greater coverage in screening for diabetic retinopathy. | Wrong study design |
| Covarrubias - 2017 | Coverage of a screening program and prevalence of diabetic retinopathy in primary care | Classification not reported |
| Lazcano-Gomez - 2017 | Neovascular Glaucoma: A retrospective review from a tertiary eye care center in Mexico | Wrong outcome |
| Morales - 2017 | Digital tool for detecting diabetic retinopathy in retinography image using gabor transform | Wrong study design |
| Navuluri - 2000 | Diabetic retinopathy screening among Hispanics in Lea County, New Mexico. | Wrong population |
| Germano - 2017 | Frequency of ocular conditions in native Brazilians from AvaÃ­ City, SÃ£o Paulo State | Wrong population |
| Leske - 2003 | Incidence of diabetic retinopathy in the Barbados Eye Studies. | Duplicate record |
| Urrutia-Aliano - 2016 | Depressive symptoms and type 2 diabetes mellitus in outpatients of an Armed Forces hospital in Lima, Peru, 2012: a cross-sectional study | Wrong outcome |
| Ribeiro - 2016 | Clinical features of diabetic patients with dry eye disease in a community in Maceio: A cross-sectional study | Classification not reported |
| Mendanha - 2016 | Risk factors and incidence of diabetic retinopathy | Wrong outcome |
| Haffner - 1989 | Effects of socioeconomic status on hyperglycemia and retinopathy levels in Mexican Americans with NIDDM. | Wrong country |
| Velázquez-González - 2015 | Detection and classification of non-proliferative diabetic retinopathy using a back-propagation neural network | Wrong outcome |
| Silva - 2015 | A comparative assessment of avoidable blindness and visual impairment in seven Latin American countries: Prevalence, coverage, and inequality | Wrong outcome |
| Limburg - 2015 | Functional low vision in adults from Latin America: Findings from population-based surveys in 15 countries | Wrong population |
| Lima-Gómez - 2001 | Choroid fundus as protective factor in the development of diabetic retinopathy. | Wrong outcome |
| Yau - 2002 | Analysis of censored and incomplete survival data using flowgraph models. | Wrong outcome |
| Moriarty - 1989 | Diabetic maculopathy in a Jamaican population. | Wrong outcome |
| Vergara - 2014 | Epidemiological and ophthalmological findings in diabetic patients examined in a general hospital | Classification not reported |
| Alvarado - 2014 | National survey of blindness and avoidable visual impairment in Honduras | Wrong population |
| Campos - 2014 | National survey on the prevalence and causes of blindness in Peru | Wrong outcome |
| Agurto - 2011 | Automatic detection of diabetic retinopathy and age-related macular degeneration in digital fundus images. | Wrong outcome |
| Castro - 2013 | Risk factors and severity of diabetic retinopathy | Wrong outcome |
| Valdés - 2013 | Clinical characteristics and frequency of chronic complications in people with newly diagnosed Type 2 Diabetes Mellitus | Duplicate record |
| Moctezuma - 2012 | Association of serum albumin with severity of diabetic retinopathy | Classification not reported |
| Oliveira - 2014 | Clinical and epidemiological profile of chronic hemodialysis patients in João Pessoa--PB. | Wrong outcome |
| Furtado - 2012 | Causes of Blindness and Visual Impairment in Latin America | Wrong study design |
| Monteiro - 2012 | Grupal rehabilitation: Expectations and perceptions of people with visual disabilities | Wrong publication type |
| Bittencourt - 2011 | Diabetic retinopathy and visual disabilities among patients in a rehabilitation program | Wrong outcome |
| Mota - 2011 | Coronary artery disease associated with low-grade albuminuria in type 2 diabetes mellitus | Classification not reported |
| Ovalle-Luna - 2019 | Prevalencia de complicaciones de la diabetes y comorbilidades asociadas en medicina familiar del Instituto Mexicano del Seguro Social. | Classification not reported |
| Connell - 1988 | Eye health. | Wrong study design |
| Cano - 2007 | Prevalence of diabetic retinopathy and barriers to uptake of eye care services by diabetic patients at the Social Security Institute Central Hospital in Asunción, Paraguay. | Wrong publication type |
| Rodrigues - 2010 | Characterization of patients with type 1 diabetes mellitus in Southern Brazil: Chronic complications and associated factors | Duplicate record |
| Hennis - 2009 | Nine-year Incidence of Visual Impairment in the Barbados Eye Studies | Wrong outcome |
| García-Alcolea - 2009 | Clinical behavior of diabetic retinopathy in Maiquetía Parish, Vargas, Venezuela 2007 | Classification not reported |
| Guedes - 2009 | Prevalence of the diabetic retinopathy in a family's health program unity | Duplicate record |
| Lesso-Zamora - 2009 | Systemic control of a diabetic population at the time of its admission to an ophthalmologic reference center | Wrong population |
| Gonçalves - 2008 | Epidemiology and regional differences of diabetic retinopathy in Pernambuco, Brazil | Duplicate record |
| Rodrigues - 2015 | Diabetes induces changes in neuroretina before retinal vessels: a spectral-domain optical coherence tomography study. | Wrong outcome |
| Walsh - 2006 | A multinational assessment of complications in type 1 diabetes: the DiaMond substudy of complications (DiaComp) level 1. | Wrong population |
| Mosenzon - 2013 | Baseline characteristics of the patient population in the Saxagliptin Assessment of Vascular Outcomes Recorded in patients with diabetes mellitus (SAVOR)-TIMI 53 trial. | Wrong study design |
| Liew - 2008 | Birth weight is not related to risk of diabetic retinopathy in type 2 diabetes: The atherosclerosis risk in communities study | Wrong population |
| Wong - 2008 | Relation between fasting glucose and retinopathy for diagnosis of diabetes: three population-based cross-sectional studies | Wrong population |
| Herrera-Bello - 2007 | Detection of chronic renal disease in diabetes mellitus in a health area | Classification not reported |
| Oliveira - 2007 | Delay in ophthalmologic examination of diabetic retinopathy patients | Wrong population |
| Sampaio - 2007 | Nephropathy and retinopathy in type 1 diabetics assisted by a universitary multiprofessional program | Duplicate record |
| Leske - 2006 | Nine-year incidence of diabetic retinopathy in the Barbados eye studies | Wrong country |
| Leitão - 2005 | Urinary albumin excretion rate is associated with increased ambulatory blood pressure in normoalbuminuric type 2 diabetic patients | Classification not reported |
| Leske - 2005 | Hyperglycemia, blood pressure, and the 9-year incidence of diabetic retinopathy: The Barbados Eye Studies | Wrong country |
| Martins - 2004 | Aerobic Bacterial Conjunctival Flora in Diabetic Patients | Wrong outcome |
| Costa - 2004 | Aggregation of features of the metabolic syndrome is associated with increased prevalence of chronic complications in Type 2 diabetes | Classification not reported |
| Villegas - 2004 | Control and chronic complications of diabetes mellitus in an Outpatient Attention Center in Medellín, Colombia, 1998-2001 | Classification not reported |
| De Souza - 2004 | Diabetic retinopathy among patients assisted by a multidisciplinary program at the University Hospital of Ribeirão Preto, São Paulo - USP | Classification not reported |
| Leske - 2004 | Four-Year Incidence of Visual Impairment: Barbados Incidence Study of Eye Diseases | Wrong outcome |
| Scheffel - 2004 | Prevalence of micro and macroangiopatic chronic complications and their risk factors in the care of out patients with type 2 diabetes mellitus | Classification not reported |
| Hennis - 2002 | Diabetes in a Carribean population: Epidemiological profile and implications | Wrong outcome |
| Gomes - 2000 | Prospective study of development of microalbuminuria and retinopathy in Brazilian IDDM patients | Wrong population |
| Villalobos - 1999 | Diabetic retinopathy and risk of blindness in Mexico: Are we doing enough? | Wrong publication type |
| Leske - 1999 | Diabetic retinopathy in a black population: The Barbados eye study | Wrong country |
| Ortiz - 2020 | Disentangling socioeconomic inequalities of type 2 diabetes mellitus in Chile: A population-based analysis | Wrong outcome |
| Gonzalez - 1994 | The level of metabolic control in low income Mexico City diabetics. The Mexico City diabetes study | Wrong outcome |
| Schachat - 1993 | Comparison of Diabetic Retinopathy Detection by Clinical Examinations and Photograph Gradings | Wrong country |
| Verdaguer - 1987 | Nonproliferative diabetic retinopathy with significant capillary nonperfusion | Wrong study design |
| Ferreira - 2020 | Causes of visual functional low vision in a tertiary ophthalmic service in ribeirão preto, são paulo, brazil | Wrong publication type |
| Brait - 2019 | Epidemiology and prevalence of diabetic retinopathy in a population assisted at world diabetes day in presidente prudente-SP | Wrong publication type |
| Do Nascimento - 2019 | Effect of high Vitamin D doses on diabetic retinopathy in patients with type 1 diabetes mellitus | Wrong publication type |
| Urrets-Zavalia - 1977 | Diabetic retinopathy | Wrong publication type |
| Aiello - 1973 | The Diabetic Retinopathy Study | Wrong publication type |
| Huarachi - 2019 | Correlation of ocular surface parameters, retinopathy and autonomic and peripheral neuropathy in type 2 diabetic patients | Wrong outcome |
| Marín - 2019 | Screening for diabetic retinopathy and other retinal diseases: A telemedicine project in Mexico | Wrong publication type |
| Rios - 2019 | Deep learning method to identify diabetic retinopathy and diabetic macular edema characteristics | Wrong publication type |
| Jesus - 2019 | Diabetes as leading cause of permanent occupational disability due to ocular diseases in the mexican institute of social security | Wrong outcome |
| Grupenmacher - 2018 | Correlations between dry eye, ocular surface findings, autonomic and sensitive neuropathy in diabetic patients with Charcot Joint Disease | Wrong publication type |
| Romero - 2018 | Diabetic retinopathy in rural population, experience in a mexican institution of ophthalmology | Wrong publication type |
| Franco - 2018 | Relationship of diabetic retinopathy in cognitive and functional state in patients with stroke | Wrong publication type |
| Portilho - 2018 | Diabetic retinopathy in participants of a health promotion program in the north/Brazil region | Wrong publication type |
| Nobre - 2018 | Transversal study of type 2 diabetes patient profile in three general ambulatories of endocrinology of a reference service | Wrong publication type |
| Gorejko - 2018 | Evaluation of the profile of patients with diabetic neuropathy at a public reference center | Wrong publication type |
| Sereday - 2008 | Chronic complications in patients with newly diagnosed type 2 diabetes. | Classification not reported |
| Carrazedo - 2018 | Proliferative diabetic retinopathy correlates with the presence of microalbuminuria in a sample of type 2 diabetic patients | Wrong publication type |
| Queiroga - 2018 | High prevalence of comorbidities and complications related to diabetes in patients linked to a care line | Wrong publication type |
| Fritzen - 2018 | High prevalence of psychiatric disorders and association with increased frequency of acute and chronic complications in pacients with type 1 diabete mellitus: Cross-sectional evaluation in the south of Brazil | Wrong publication type |
| Flores-González - 2017 | Telemedicine for detection of ocular diseases capable to cause blindness in Mexico | Wrong outcome |
| Valencia-Santiago - 2017 | Staging of diabetic retinopathy in a sample of patients in Mexico through a reading center | Wrong publication type |
| Camacho-Martinez - 2017 | Main visual disorders of the geriatric patient: Report of an ophthalmologic reference center in the north of Mexico | Wrong publication type |
| Cuadras - 2017 | Quantification of VEGF in peripheral blood as a marker of activity in diabetic retinopathy | Wrong publication type |
| Singh - 2017 | Microalbuminuria as a reliable indicator of diabetic retinopathy in childhood onset diabetes mellitus | Wrong outcome |
| Acuna - 2017 | Prevalence of complications in patients with diabetes mellitus in Colombia | Wrong publication type |
| Mowatt - 2017 | Audit of the diabetic retinopathy screening (DRS) at the UHWI: saving vision | Wrong publication type |
| De Andrade - 2016 | Risk factors and the incidence of patients with diabetic retinopathy in patients of eyes institute of Goiânia; Goiânia-go | Duplicate record |
| Jimenez-Corona - 2016 | Prevalence of retinopathy and macular edema and evaluation of associated risk factors in patients with type 2 diabetes of short duration | Wrong publication type |
| Gomes - 2016 | Does parity worsen diabetes-related chronic complications in women with type 1 diabetes? | Classification not reported |
| Sugrim - 2016 | The prevalence and treatment requirements of patients with diabetic retinopathy at the Georgetown public hospital eye clinic | Wrong publication type |
| Holmes - 2016 | Prevalence of proliferative diabetic retinopathy 15-25 years post diagnosis of type 2 diabetesmellitus and its association with traditional risk factors for cardiovascular disease in barbados | Wrong country |
| Lopez-Rubio - 2015 | Non-mydriatic fundus photography vs. Clinical ophthalmoscopy for diabetic retinopathy in patients with early diabetes diagnosis | Wrong publication type |
| Jimenez-Baez - 2015 | Early diagnosis of diabetic retinopathy in primary care | Duplicate record |
| Rosas-Romero - 2015 | A method to assist in the diagnosis of early diabetic retinopathy: Image processing applied to detection of microaneurysms in fundus images | Wrong outcome |
| Viteri - 2014 | Panretinal photocoagulation for retina disease | Wrong study design |
| Sakata - 2014 | Prevalence and causes of visual impairment and blindness in an urban population: The south brazilian bocaiuva study lisandro sakata | Wrong publication type |
| Schellini - 2014 | Prevalence of diabetes and diabetic retinopathy in a Brazilian population | Wrong outcome |
| Gomez - 2014 | Clinical impact of sensor-augmented insulin pump (SAP) therapy in type 1 diabetes long-term related complications in Colombia | Wrong publication type |
| Mowatt - 2013 | Diabetic retinopathy and its risk factors at the University Hospital in Jamaica | Classification not reported |
| Viégas - 2011 | Prevalence of osteoporosis and vertebral fractures in postmenopausal women with type 2 diabetes mellitus and their relationship with duration of the disease and chronic complications | Wrong outcome |
| Colli - 2011 | Mutation H63D in the HFE gene confers risk for the development of type 2 diabetes mellitus but not for chronic complications | Wrong study design |
| Kramer - 2011 | Afternoon blood pressure increase: A blood pressure pattern associated with microvascular complications in type 2 diabetes mellitus | Wrong outcome |
| Dias - 2010 | Epidemiologic profile and level of knowledge among diabetic patients about diabetes and diabetic retinopathy | Classification not reported |
| Rodrigues - 2010 | Masked hypertension, nocturnal blood pressure and retinopathy in normotensive patients with type 1 diabetes | Classification not reported |
| Kramer - 2009 | Late afternoon blood pressure increase is associated with diabetic retinopathy in normotensive type 2 diabetes mellitus patients | Wrong outcome |
| Leite - 2009 | Cataract remains an important cause of blindness in Campinas, Brazil | Wrong outcome |
| Wobeto - 2007 | Haptoglobin polymorphism and diabetic retinopathy in Brazilian patients | Classification not reported |
| León-Morales - 2005 | Auditory impairment in patients with type 2 diabetes mellitus | Classification not reported |
| Santos - 2003 | Diabetic retinopathy in Euro-Brazilian type 2 diabetic patients: Relationship with polymorphisms in the aldose reductase, the plasminogen activator inhibitor-1 and the methylenetetrahydrofolate reductase genes | Duplicated population |
| Quiroz-Mercado - 2002 | Human lymphocyte antigen DR7 protects against proliferative retinopathy with type II diabetes mellitus | Wrong study design |
| Moreira - 2001 | Use of optical coherence tomography (OCT) and indirect ophthalmoscopy in the diagnosis of macular edema in diabetic patients | Wrong population |
| Wilks - 2001 | Management of diabetes mellitus in three settings in Jamaica | Wrong outcome |
| Urrets-Zavalia - 1977 | Diabetic retinopathy | Wrong publication type |
| Rodríguez-Villalobos - 2005 | [Diabetic retinopathy: twelve-year incidence and progression]. | Wrong population |
| Malone - 2022 | Retinopathy During the First 5 Years of Type 1 Diabetes and Subsequent Risk of Advanced Retinopathy. | Wrong population |
| Muzy - 2021 | [Prevalence of diabetes mellitus and its complications and characterization of healthcare gaps based on triangulation of studies]. | Wrong outcome |
| Asenjo-Alarcón - 2022 | Chronic microvascular complications in users with type 2 diabetes mellitus from an Andean city in Peru | Wrong population |
| Longa-López - 2022 | Prevalence and factors associated with retinopathy in patients of the integral diabetes program of the San Genaro de Villa Chorillos health center, Lima-Peru | Duplicate record |
| Vivas-Giraldo - 2021 | Characterization of diabetic retinopathy in a screening program in Medellin, Colombia, in 2018 | Wrong outcome |
| Marcano-Caraballo - 2022 | Epidemiological features of patients ascribed to a primary care cardiovascular health program in Santiago, Chile | Wrong outcome |
| Tang - 2021 | Detection of Diabetic Retinopathy from Ultra-Widefield Scanning Laser Ophthalmoscope Images: A Multicenter Deep Learning Analysis. | Wrong population |
| Bergonsi - 2021 | Glycated Hemoglobin and Blood Pressure Levels in Adults With Type 2 Diabetes: How Many Patients Are on Target? | Wrong study design |
| Joshi - 2021 | A retrospective study of causes of visual impairment and use of low vision devices in the low vision clinic in Trinidad and Tobago. | Classification not reported |
| Vivas-Giraldo - 2021 | Characterization of diabetic retinopathy in a screening program in Medellin, Colombia, in 2018 | Duplicate record |
| Bravo - 2022 | Diabetic Retinopathy and Diabetic Macular Edema in a population of Antioquia. Cross-sectional study | Classification not reported |
| Ortiz-Basso - 2022 | Prevalence of diabetic retinopathy in a rural area of Argentina | Duplicate record |
| Galvão - 2021 | Prevalence and Risk Factors of Diabetic Retinopathy in Patients with Diabetes Seen by Unscheduled Demand: a Cross-sectional Study | Duplicate record |
| Barrera-Guarderas - | Diabetic retinopathy: long-term follow-up of Ecuadorian patients with type 2 diabetes in primary care | Wrong outcome |
| Longa-López - 2022 | PREVALENCE AND FACTORS ASSOCIATED WITH RETINOPATHY IN PATIENTS OF THE INTEGRAL DIABETES PROGRAM OF THE SAN GENARO DE VILLA CHORILLOS HEALTH CENTER, LIMA-PERU | Wrong outcome |
