# Supplementary material for: Prevalence and incidence of diabetic retinopathy in patients with diabetes of Latin America and the Caribbean: A systematic review and meta-analysis
Source: PLoS One. 2024 Apr 4;19(4):e0296998. doi: 10.1371/journal.pone.0296998 (PMC10994322; doi:10.1371/journal.pone.0296998)
Supplement: S4 Table — (DOCX) [file pone.0296998.s008.docx]

| Supplementary material 4. Characteristics of the included studies assessing the incidence of diabetic retinopathy in Latin America and the Caribbean (n=1). | | | | | | | | | | | |
| --- | --- | --- | --- | --- | --- | --- | --- | --- | --- | --- | --- |
| Author - year | Country | Follow-up | Setting | Sample size | DM type | Age (mean ± SD years), male (%), diabetes time (mean ± SD years), A1c (mean ± SD %) | Diabetic retinopathy diagnostic method | Diabetic retinopathy accumulative incidence | | | Quality score (Max. 9) |
|  |  |  |  |  |  |  |  | Total | NPDR | PDR |  |
| Leske - 2006 | Barbados | 9 years | Community | 436 | T1DM/ T2DM | Age: 57.6 ± 9.4 | ETDRS | 39.6% | General: NR | 2.6% | 7 |
|  |  |  |  |  |  | Male: 36.7% |  |  | Mild: NR |  |  |
|  |  |  |  |  |  | Diabetes time: 7.3 ± 7.5 |  |  | Moderate: NR |  |  |
|  |  |  |  |  |  | HbA1c: 10.8 ± 3.3 |  |  | Severe: NR |  |  |
| DM: Diabetes Mellitus, T1DM: Type 1 Diabetes Mellitus, T2DM: Type 2 Diabetes Mellitus, NDPR: Non-proliferative diabetic retinopathy, PDR: Proliferative diabetic retinopathy, ETDRS: Early Treatment Diabetic Retinopathy Study. | | | | | | | | | | | |
