# Supplementary material for: Prevalence and incidence of diabetic retinopathy in patients with diabetes of Latin America and the Caribbean: A systematic review and meta-analysis
Source: PLoS One. 2024 Apr 4;19(4):e0296998. doi: 10.1371/journal.pone.0296998 (PMC10994322; doi:10.1371/journal.pone.0296998)
Supplement: S5 Table — (DOCX) [file pone.0296998.s009.docx]

| Supplementary material 7. Meta-regression models of diabetic retinopathy in Latin America and the Caribbean countries. | | | | | | | | | |
| --- | --- | --- | --- | --- | --- | --- | --- | --- | --- |
|  |  |  | Crude | | | | Adjusted | | |
|  | Variable | n studies | β | 95% CI | p value | Adjusted R^2^ | β | 95% CI | p value |
| T1DM | |  |  |  |  |  |  |  |  |
|  | Mean age (years) | 8 | **0.013** | **0.002 to 0.025** | **0.031** | 57.8 |  |  |  |
|  | Diabetes time (years) | 8 | 0.012 | -0.001 to 0.025 | 0.054 | 54.2 |  |  |  |
|  | Risk of bias score | 9 | 0.016 | -0.060 to 0.083 | 0.715 | -44.5 |  |  |  |
|  | Publication year | 9 | 0.010 | -0.010 to 0.029 | 0.270 | -96.7 |  |  |  |
| T2DM | |  |  |  |  |  |  |  |  |
|  | Mean age (years) | 18 | 0.005 | -0.017 to 0.026 | 0.643 | -5.8 | -0.007 | -0.021 to 0.006 | 0.224 |
|  | Diabetes time (years) | 8 | **0.024** | **0.009 to 0.039** | **0.008** | **100.0** | **0.032** | **0.011 to 0.053** | **0.010** |
|  | Risk of bias score | 19 | 0.002 | -0.088 to 0.093 | 0.955 | -7.3 |  |  |  |
|  | Publication year | 19 | -0.009 | -0.025 to 0.007 | 0.250 | 3.8 |  |  |  |
| T1DM: Type 1 diabetes mellitus. T2DM: type 2 diabetes mellitus.  In bold significant p-values (p<0.05) | | | | | | | | | |
