# Supplementary material for: Prevalence and incidence of diabetic retinopathy in patients with diabetes of Latin America and the Caribbean: A systematic review and meta-analysis
Source: PLoS One. 2024 Apr 4;19(4):e0296998. doi: 10.1371/journal.pone.0296998 (PMC10994322; doi:10.1371/journal.pone.0296998)
Supplement: S6 Table — (DOCX) [file pone.0296998.s010.docx]

| Supplementary material 10. Risk of bias of included studies using the JBI Critical Appraisal Tool for prevalence studies. | | | | | | | | | | |
| --- | --- | --- | --- | --- | --- | --- | --- | --- | --- | --- |
| Author - year | Was the sample frame appropriate to address the target population? | Were study participants sampled in an appropriate way? | Was the sample size adequate? | Were the study subjects and the setting described in detail? | Was the data analysis conducted with sufficient coverage of the identified sample? | Were valid methods used for the identification of the condition? | Was the condition measured in a standard, reliable way for all participants? | Was there appropriate statistical analysis? | Was the response rate adequate, and if not, was the low response rate managed appropriately? | Quality score (Max. 9) |
| Arenas-Cavalli - 2022 | No | No | Yes | No | Yes | Yes | Yes | Yes | Yes | 6 |
| Ortiz-Basso - 2022 | No | No | Yes | Yes | Yes | Yes | Yes | Yes | Yes | 7 |
| Galvão - 2021 | No | No | No | Yes | Yes | Yes | Yes | Yes | Unclear | 5 |
| Graue-Hernandez - 2020 | No | Yes | Yes | Yes | Yes | Yes | Yes | Yes | Unclear | 7 |
| Abalem - 2020 | No | Yes | No | Yes | Yes | Yes | Yes | No | Unclear | 5 |
| Ben - 2020 | No | No | No | Yes | Yes | Yes | Yes | No | Yes | 5 |
| Adrianzén - 2019 | No | No | Yes | Yes | Yes | Yes | Yes | Yes | Yes | 7 |
| Acevedo - 2019 | Yes | Yes | Yes | No | No | Yes | Yes | Yes | No | 6 |
| Avendaño-Veloso - 2019 | No | Yes | Yes | No | No | Yes | Yes | No | Yes | 5 |
| Lopez-Ramos - 2018 | Yes | Yes | Yes | No | Yes | Yes | Yes | Yes | Yes | 8 |
| Nunes Melo - 2018 | No | Yes | Yes | No | Yes | Yes | Yes | No | Yes | 6 |
| Lima - 2018 | Yes | No | No | Yes | No | Yes | Yes | No | No | 4 |
| Lopez-Star - 2018 | Yes | Yes | Yes | No | Unclear | Yes | Yes | Yes | Yes | 7 |
| Rosses - 2017 | No | No | No | Yes | Yes | Yes | Yes | No | Yes | 5 |
| Mendoza-Herrera - 2017 | Yes | No | Yes | Yes | No | Yes | Yes | No | No | 5 |
| Flores-Mena - 2017 | No | Yes | No | Yes | Unclear | Yes | Yes | No | Yes | 5 |
| Rodriguez - 2016 | No | Yes | Yes | Yes | No | Yes | Yes | Yes | Yes | 7 |
| Minderhoud - 2016 | Yes | Yes | Yes | No | No | Yes | Yes | No | Yes | 6 |
| Malerbi - 2015 | No | No | Yes | Yes | Yes | Yes | Yes | No | Yes | 6 |
| Cepeda-Nieto - 2015 | No | No | No | Yes | Yes | Yes | Yes | No | Unclear | 4 |
| Valdés - 2013 | No | Unclear | No | Yes | Yes | Yes | Yes | No | Unclear | 4 |
| Alcaraz - 2013 | No | Yes | No | Yes | Yes | Yes | Yes | Yes | Yes | 7 |
| Polack - 2012 | Yes | Yes | Yes | No | Unclear | Yes | Yes | Yes | No | 6 |
| Perera - 2011 | Yes | Yes | No | No | No | Yes | Yes | No | Yes | 5 |
| Almeida - 2011 | No | Yes | No | No | Unclear | Yes | Yes | No | Unclear | 3 |
| Carrillo-Alarcón - 2011 | No | Yes | No | Yes | No | Yes | Yes | Yes | Yes | 6 |
| Villena - 2011 | No | Yes | Yes | No | Unclear | Yes | Yes | No | Yes | 5 |
| Rodrigues - 2010 | Yes | No | Yes | Yes | Yes | Yes | Yes | No | Yes | 7 |
| Preti - 2010 | No | Yes | No | Yes | Yes | Yes | Yes | No | Yes | 6 |
| Sawitzki - 2010 | Yes | Yes | No | Yes | Yes | Yes | Yes | No | Yes | 7 |
| Prado-Serrano - 2009 | No | Yes | Yes | Yes | No | Yes | Yes | No | No | 5 |
| Esteves - 2009 | No | No | Yes | Yes | Yes | Yes | Yes | Yes | Yes | 7 |
| Gonçalves - 2008 | Yes | No | Yes | No | No | Yes | Yes | No | Yes | 5 |
| Lisboa - 2008 | No | Yes | No | Yes | Yes | Yes | Yes | No | Yes | 6 |
| Sampaio - 2007 | No | No | No | Yes | Yes | Yes | Yes | No | Yes | 5 |
| Licea - 2006 | No | Yes | No | Yes | Yes | Yes | No | No | Yes | 5 |
| Leske - 2006 | Yes | Yes | Yes | No | No | Yes | Yes | Yes | Yes | 7 |
| Santos - 2005 | No | Yes | No | Yes | Yes | Yes | Yes | No | Yes | 6 |
| Crespo - 2004 | No | Yes | No | No | No | Yes | Yes | No | Yes | 4 |
| Pereira - 2004 | No | Unclear | No | Yes | No | Yes | Yes | No | Unclear | 3 |
| Alvarenga - 2003 | Yes | No | Yes | No | Yes | Yes | Yes | No | Yes | 6 |
| Lima-Gómez - 2001 | Yes | No | Yes | No | Unclear | Yes | Yes | No | Yes | 5 |
| Lima-Gómez - 2001 | No | Yes | Yes | No | No | Yes | Yes | No | Yes | 5 |
| Villalpando - 1997 | Yes | Yes | No | Yes | Yes | Yes | Yes | Yes | No | 7 |
