# Supplementary material for: Prevalence and incidence of diabetic retinopathy in patients with diabetes of Latin America and the Caribbean: A systematic review and meta-analysis
Source: PLoS One. 2024 Apr 4;19(4):e0296998. doi: 10.1371/journal.pone.0296998 (PMC10994322; doi:10.1371/journal.pone.0296998)
Supplement: Attachment — Submitted filename: Response diabetic retinopathy.docx [file pone.0296998.s011.docx]

**Response to the reviewers**

**Reviewer comments**

**Comment**: Thanks for the opportunity to review the manuscript again. We can find that many improvements were registered. The authors appropriately reviewed and addressed the recommendations in a new version of the manuscript. However, some relevant methodological issues need to be addressed to avoid the risk of bias.

In order to have consistency, the search strategy must be written according to the JBI methodology for systematic review. There seems to be little understanding of the difference between an information source and a database. So far, it needs to be more clarity in the description of the search strategy and not in how the search was conducted. The text conveys that only journals in the indexing based on the Web of Science were accessed. See at the topic "Literature search and study selection": "A systematic search was performed in six databases: Web of Science (WoS)/Core Collection, WoS/MEDLINE, WoS/Scielo, Scopus, PubMed and Embase. It would be an inconsistency since texts duplicated in multiple databases were excluded. See the JBI Manual, the Systematic Review chapter, for a better presentation of the sources accessed.

Reply: Thank you very much for your comment, we have checked the JBI manual, chapter 5: systematic reviews of prevalence and incidence, section 5.5.5 search strategy. For clarification, we have changed the paragraph: “*A systematic search was performed in six information sources: Web of Science (WoS)/Core Collection, WoS/MEDLINE, WoS/Scielo, Scopus, PubMed and Embase until January 16, 2023*.” The search strategy is detailed in S2 table.

**Comment**: Throughout the method section's text, the JBI's name is recorded in its previous form. Please check the link (https://jbi.global/news/article/revealed-new-jbi) for the current name and the justification for its suitability. JBI's new name and logo signify its evolution and capture how the organization is known internationally today. That is, as 'JBI'—for example, JBI critical appraisal tools and JBI methodology.

**Reply:** Thanks for the comment, we have changed to “JBI” throughout the manuscript.
