# Supplementary material for: Prevalence and incidence of diabetic retinopathy in patients with diabetes of Latin America and the Caribbean: A systematic review and meta-analysis
Source: PLoS One. 2024 Apr 4;19(4):e0296998. doi: 10.1371/journal.pone.0296998 (PMC10994322; doi:10.1371/journal.pone.0296998)
Supplement: Attachment — Submitted filename: Response diabetic retinopathy.docx [file pone.0296998.s012.docx]

**Response to the reviewers**

**Reviewer comments**

**Comment**: After a new reading, only one inconsistency in the writing was identified: a methodological mistake for systematic reviews according to the JBI methodology.

The response to the peer review recommendation states that “A systematic search was performed in six information sources: Web of Science (WoS)/Core Collection, WoS/MEDLINE, WoS/Scielo, Scopus, PubMed and Embase until January 16, 2023. ”

The Core Collection is a source of information with Web of Science as one of the journal indexing bases. Science Direct is the source of information, with Scopus as one of the journal indexing bases. Medline is a journal indexing database that can be accessed through the PubMed Library. It is not possible to access journals indexed in MedLine through WoS.

The same information needs to be reviewed concerning collecting journals indexed in SciELO.

In this sense, the information in the manuscript does not correspond to what PONE readers can use in the future. However, when conducting a review whose starting point is to include only journals accessed by the Core Collection source of information or journals from the WoS index database, it produces a bias in the analysis of evidence.

**Reply**: Thank you for your valuable feedback. In response to your suggestion, we have opted to revise the term "*sources of information*" to simply "*sources*," aligning with Cochrane's general usage in describing the search scope of systematic reviews. Regarding Web of Science, it is important to note that it comprises a collection of databases. Although a general search is possible across all databases included in Web of Science without specifying a particular database, we recognize the importance of adapting the search strategy for each database to ensure precision. For instance, when searching MEDLINE through Web of Science, MeSH terms are employed, whereas Core Collection necessitates a different approach without the use of MeSH terms. Therefore, the search strategies for Core Collection, MEDLINE and Scielo are different. Regarding MEDLINE in Web of Science, it is available from 1950 to the present. Once again, we appreciate your thorough review and constructive input, which will significantly enhance the accuracy and transparency of our systematic review.
